# Urinary metals in a spontaneous canine model of calcium oxalate urolithiasis

**DOI:** 10.1371/journal.pone.0176595

**Published:** 2017-05-03

**Authors:** Eva Furrow, Molly E. McCue, Jody P. Lulich

**Affiliations:** 1 Department of Veterinary Clinical Sciences, College of Veterinary Medicine, University of Minnesota, St. Paul, Minnesota, United States of America; 2 Department of Veterinary Population Medicine, College of Veterinary Medicine, University of Minnesota, St. Paul, Minnesota, United States of America; University of Louisville School of Medicine, UNITED STATES

## Abstract

Calcium oxalate urolithiasis is a common and painful condition in people. The pathogenesis of this disease is complex and poorly understood. Laboratory animal and *in vitro* studies have demonstrated an effect of multiple trace metals in the crystallization process, and studies in humans have reported relationships between urinary metal concentrations and stone risk. Dogs are a spontaneous model of calcium oxalate urolithiasis, and the metal content of canine calcium oxalate stones mirrors that of human stones. The aim of this study was to test for a relationship between urinary metals and calcium oxalate urolithiasis in dogs. We hypothesized that urinary metals would differ between dogs with and without calcium oxalate urolithiasis. Urine from 122 dogs (71 cases and 51 stone-free controls) was analyzed for calcium and 12 other metals. The cases had higher urinary calcium, copper, iron, and vanadium and lower urinary cobalt. Higher urinary vanadium in the cases was associated with being fed a therapeutic stone-prevention diet. Urinary calcium had a strong positive correlation with strontium and moderate positive correlations with chromium, nickel, and zinc. The results of this study complement the findings of similar human studies and suggest a potential role of trace metals in calcium oxalate urolithiasis. Further investigation into how trace metals may affect stone formation is warranted.

## Introduction

Urolithiasis is a significant health problem, with a lifetime incidence of 12% in men and 6% in women [1, 2]. Preventative treatments such as increasing water intake and thiazide diuretics can significantly reduce risk [3], but the overall stone recurrence rate is high at 50% by 5 years [4]. Approximately 75% of kidney stones are composed of calcium oxalate (CaOx) [5,6], and idiopathic hypercalciuria (high urinary calcium) is the most common metabolic abnormality in stone patients [7]. The prevalence of CaOx urolithiasis is increasing; however, specific environmental and dietary exposures involved have been difficult to identify [8]. Novel research approaches are needed to uncover etiopathologic factors in stone development.

While most laboratory animals seem relatively resistant to forming CaOx stones, dogs develop them naturally. As with humans, canine CaOx stones are common, recurrent, and associated with hypercalciuria [9,10]. The prevalence of CaOx urolithiasis is also on the rise in dogs, and it is hypothesized that shared environmental risk factors are responsible [11]. Dogs are one of the top sentinel animals for detecting environmental toxins [12] and thus offer a model for studying the role of environmental exposures in stone development.

Several metals have been implicated in lithogenesis in humans and laboratory animals, but the relationships between individual metals and stone risk are not well understood [13]. Zinc, strontium, and iron are the most abundant metals in kidney stones, totaling close to 1% of the stone composition, and these metals are detected at greater quantities in calcium-containing stones than other stone types [14]. A recent study discovered that the metal content of canine CaOx stones closely mirrors that of human CaOx stones with relatively high zinc, strontium, and iron [15].

Additionally, metals that are not commonly detected within uroliths can still play an important role in the disease. Cadmium is a classic example. It is an environmental toxin that causes renal damage and alters calcium homeostasis. Low level Cd exposure in people is associated with risk for osteoporosis, hypercalciuria and nephrolithiasis, without other overt signs of toxicity [16, 17]. Cadmium concentrations in canine blood can detect environmental cadmium contamination [18].

The objective of this study was to evaluate the relationship between urinary metals, urinary calcium, and CaOx urolithiasis in a large population of dogs from diverse breed groups. We hypothesized that urinary metal concentrations would differ between dogs with and without CaOx urolithiasis. The findings of this study parallel data from human stone formers and stress the need for further investigation into the role of metals in lithogenesis in both people and companion animals.

## Materials and methods

### Ethics statement

Written informed consent was obtained from owners of study participants, and the study protocol was approved by the University of Minnesota Institutional Animal Care and Use Committee (Protocol #1509-33019A).

### Animals

The study took place at the Veterinary Medical Center, University of Minnesota (VMC UMN). Dogs were recruited from February 2011 to March 2014. This was a cross-sectional study, and data was collected at a single time point for each dog. Cases were dogs with a history of uroliths composed of CaOx (≥70% of the central core), as determined by standard stone analysis at the Minnesota Urolith Center (polarizing light microscopy and infrared spectroscopy). The majority of the cases were recruited when presenting for stone removal procedures (active stone disease), but a subset of cases was recruited at variable time points after historical stone removal. Controls were ≥ 8 yo and had no history of CaOx uroliths, no evidence of current radiopaque uroliths on screening abdominal radiographs, and no CaOx crystalluria on urinalysis. Roughly two thirds of the study population comprised a breed- and sex-matched case-control cohort that was also enrolled in another study on urinary calcium in three breeds with the greatest risk for CaOx urolithiasis (Bichons Frise, Miniature Schnauzers, and Shih Tzus) [10]. No breed restrictions were placed on the other dogs recruited.

Dogs were excluded from the study if they had received glucocorticoids within the past week or another drug with known effects on urinary calcium excretion (i.e. furosemide, thiazide diuretics, levothyroxine, theophylline, potassium citrate) within the past 24 hours. Dogs were also excluded if they had a clinical diagnosis of a disease that alters urinary calcium excretion (i.e. hyperparathyroidism, hypercalcemia of malignancy, hyperadrenocorticism, diabetes mellitus, osteolytic disease, granulomatous disease). The current diet was recorded for each dog and classified into one of three groups: the therapeutic stone-prevention diet Hill’s Prescription Diet u/d Canine (Hill’s Pet Nutrition Inc.), the therapeutic stone-prevention diet Royal Canin Veterinary Diet canine urinary SO (Waltham Centre for Pet Nutrition), or other (diets not formulated for the prevention of stone disease; this group comprised a large variety of diets).

### Urine measurements

Owners were instructed to withhold food, but not water, for 12–18 hours prior to sample collection. The dogs urinated at least once prior to sample collection such that the research urine sample was never the first micturition of the day. Urine was collected by free catch, cystocentesis, or catheterization. Unfiltered 1–3 ml urine aliquots were immediately analyzed for calcium (spectroscopy and the calcium sensitive dye Arsenazo II) and creatinine (modified Jaffe procedure) [19,20]. A 4 ml urine aliquot was filtered through a 0.22 μm polyethersulfone membrane (MJ Research, Inc.), stored at -80°C, and analyzed for metals within 18 months. Urinary metal analysis was performed using inductively coupled plasma mass spectrometry [21]. Twelve metals were included in the analysis: barium (Ba), cadmium (Cd), cobalt (Co), chromium (Cr), copper (Cu), iron (Fe), manganese (Mn), nickel (Ni), rubidium (Rb), strontium (Sr), vanadium (V), and zinc (Zn). Spot urinary element-to-creatinine ratios (e.g. Ca/Cre, Ba/Cre, Cd/Cre) were calculated for each dog and are reported in mg/g for urinary Ca/Cre and μg/g for the other metals.

### Statistical analysis

Ages are reported as mean ± standard deviation and were compared between cases and controls with a Student’s t-test. Weight did not follow a normal distribution, is reported as median (range), and was compared between cases and controls with a Wilcoxon rank sum test. Sex proportions (males versus females) between cases and controls were compared with a Pearson’s chi-squared test. The data distribution for the urinary element-to-creatinine ratios were visually inspected with Q-Q plots. The majority of the measurements did not follow a normal distribution, thus non-parametric tests and transformations were used in the statistical analysis. Wilcoxon rank sum tests were used to compare measurements between case and control groups and for cases with and without uroliths present at the time of urine sampling. The Benjamini-Hochberg procedure was used to determine significance with a false discovery rate of 0.1; those variables that met this level of significance were analyzed further with multiple regression. For regression analyses, data was log-transformed to create a more normal distribution. Zero values were present for some of the urinary metal measurements. To manage this, the minimum non-zero value was added to each dog’s measurement for that metal such that the entire group was shifted up by the minimum value prior to log-transformation, and the data set was effectively censured at the minimum value. Regression analyses were performed for the log-transformed urinary element-to-creatinine ratios with stone status (CaOx case versus control), breed (Miniature Schnauzer, Bichon Frise, Shih Tzu, or other [breeds with n < 10 were combined]), log-transformed weight (in kg), age at the time of urine sampling (in years), and sex (male versus female) as predictors. Reduced models were generated with stepwise selection of predictors in both forward and backward directions. Akaike Information Criterion (AIC) scores were calculated as a measure of model fit, and the model with the lowest AIC was selected for the reduced model. The significance of each model predictor was assessed using Type II tests (ANCOVA). For each urinary metal found to have a relationship with stone status in the reduced model, multivariate regression were performed with the urinary element-to-creatinine ratios as the outcome and diet (Hill’s u/d Canine, Royal Canin SO, and other) and active stone disease (versus historical) as predictors. Correlation coefficients between element-to-creatinine ratios were calculated for all possible pairs. All analyses were performed using the R software for statistical computing (http://www.R-project.org/), and a p-value <0.05 was considered significant.

## Results

### Study participant characteristics

One hundred and twenty-two dogs (71 cases and 51 controls) were enrolled in the study. The most common breeds were the Miniature Schnauzer (50), Bichon Frise (22), and Shih Tzu (13). See Table 1 for additional breeds, clinical characteristics, and diets of the study participants; detailed data is also provided in S1 File. The control group was significantly older than the case group (p = 0.0056). Weight did not differ significantly between cases and controls (p = 0.18). There were more males in the case group (80% of cases versus 53% of controls, p = 0.0025), and all except for three dogs (1 male case, 1 female case, and 1 male control) were neutered. Fifty-seven cases (80%) had CaOx cystoliths at the time of sample collection (active stone disease) and had their stones removed later that same day. The remaining 14 cases had a history of prior CaOx urolithiasis. Forty-six of the cases (65%) had suffered from recurrent CaOx uroliths (median 2 stone episodes, range 1–5). Twenty-seven of the cases (38%) were on therapeutic stone-prevention diets; in all cases, these diets were prescribed after one or more episodes of CaOx urolithiasis. A single control was on a therapeutic stone-prevention diet because a household dog had a history of CaOx urolithiasis, and the owner elected to feed both dogs the same diet.

**Table 1:** Characteristics of the 122 study dogs. Cases are dogs with confirmed CaOx urolithiasis, and controls are stone-free as determined by abdominal radiography. Age at the time of urine sampling is reported as mean ± standard deviation, and weight is reported as median (range).

Stone Status	Breed	Weight	Sex	Age	Diet
**Cases** **(71 dogs)**	22 Miniature Schnauzers 11 Mixed breed dogs 10 Bichons Frise 7 Shih Tzus 5 Lhasa Apsos 3 Yorkshire Terriers 2 Rat Terriers 1 each: Chihuahua, Cocker Spaniel, Fox Terrier, Havanese, Jack Russell Terrier, Kerry Blue Terrier, Maltese, Pomeranian, Pug, Shetland Sheepdog, Welsh Terrier	8.8 (2.9–23.0) kg	57 M, 14 F	9.0 ± 2.8 yo	17 Hill’s u/d 11 Royal Canin SO 43 Other
**Controls** **(51 dogs)**	28 Miniature Schnauzers 12 Bichons Frise 6 Shih Tzus 3 Standard Schnauzer 1 each: Mixed breed, Miniature Poodle	8.2 (3.0–18.3) kg	27 M, 24 F	10.2 ± 1.9 yo	1 Royal Canin SO 40 Other

### Urinary metals and CaOx stone status

**Fig 1:**
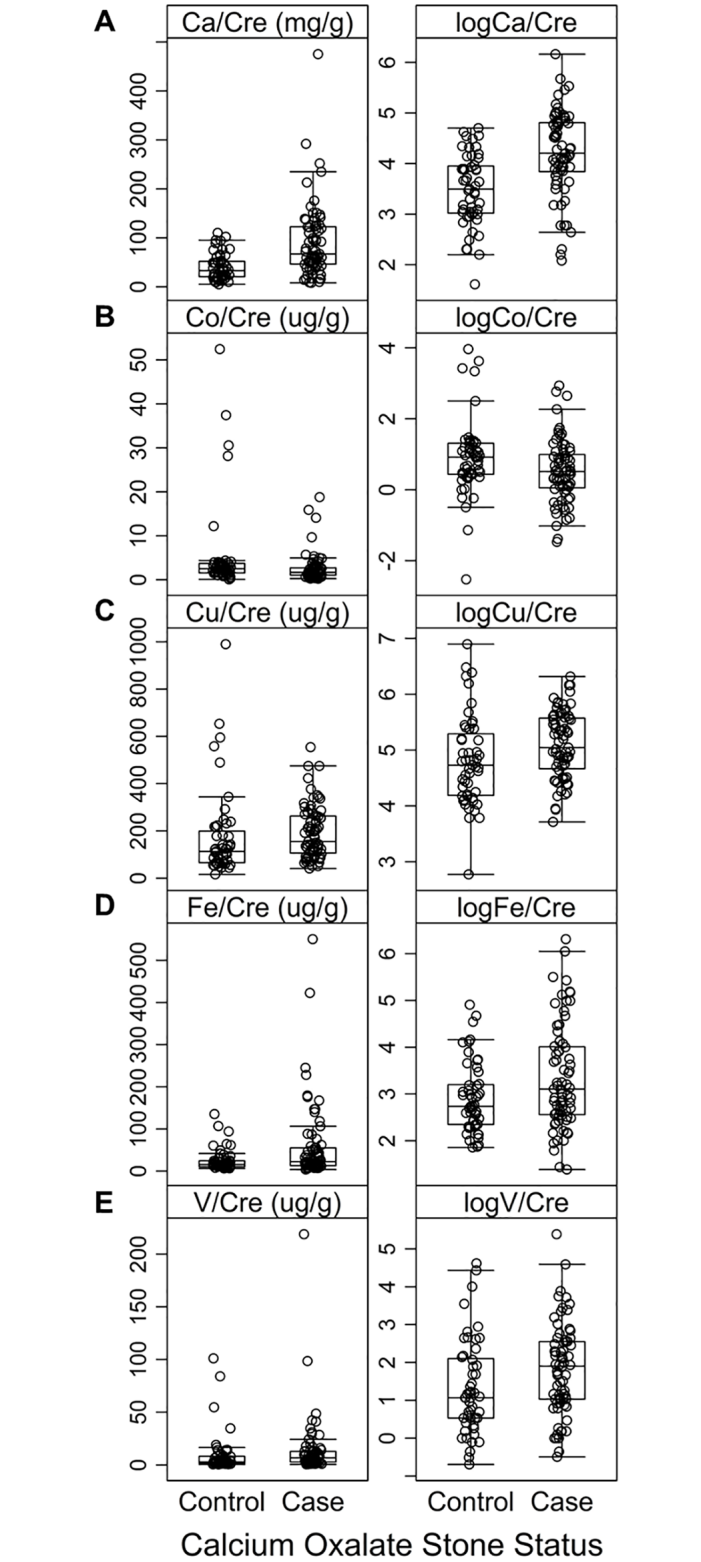
Box and whisker plots of urinary A) calcium-to-creatinine (Ca/Cre, mg/g), B) cobalt-to-creatinine (Co/Cre, μg/g), C) copper-to-creatinine (Cu/Cre, μg/g), and D) iron-to-creatinine (Fe/Cre, μg/g), and E) vanadium-to-creatinine (V/Cre, μg/g) ratios for dogs with a history of CaOx stones (cases) and stone-free dogs (controls). The boxes represent the interquartile range (25^th^– 75^th^ percentile), the horizontal line within the boxes represents the median, and the whisker bars extend to 1.5 times the interquartile range. Raw and log-transformed data are shown.

The median element-to-creatinine ratios for the case and control groups and p-values for the Wilcoxon rank sum test are reported in Table 2. Five urinary variables met the significance criteria and were selected for multivariable regression analysis: Ca/Cre, Co/Cre, Cu/Cre, Fe/Cre, and V/Cre. The results for the reduced models for each of these five urinary outcomes are reported in Table 3; full models are provided in S1 Table. For Ca/Cre, a CaOx case stone status was a strong positive predictor, and male sex was a negative predictor. For Co/Cre, stone status was the sole predictor in the reduced model with CaOx case status having a negative effect. Stone status as a CaOx case, age, and the Miniature Schnauzer breed all had a positive relationship with Cu/Cre, with stone status having the strongest effect. The models for Fe/Cre and V/Cre revealed positive relationships between these metals and being a CaOx case; stone status was the sole significant predictor for both. When the cases were analyzed alone in regressions with diet and active stone disease as predictors (S2 Table), the Hill’s u/d diet was found to have a negative association with urinary Fe/Cre (estimate = -0.90, p = 0.0042) and a positive association with urinary V/Cre (estimate = 0.74, p = 0.032). Diet did not have a significant effect on urinary Ca/Cre, Co/Cre, or Cu/Cre, and active versus historical stone disease did not have a significant relationship with any of the five urinary metals tested. The urinary Ca/Cre, Co/Cre, Cu/Cre, Fe/Cre, and V/Cre measurements in cases and controls are displayed in Fig 1.

**Table 2:** Urinary element-to-creatinine ratios in dogs with CaOx urolithiasis (cases) and stone-free controls. Values are reported in mg/g for urinary Ca/Cre and μg/g for the other urinary element-to-creatinine ratios.

Variable	Controls, Median (IQR)	Cases, Median (IQR)	Raw p-value
**Ca/Cre**	**33 (21–32)**	**67 (47–123)**	1.3E-06*
**Cu/Cre**	**113 (66–200)**	**155 (107–263)**	0.011*
**Co/Cre**	**2.5 (1.5–3.7)**	**1.7 (1.1–2.7)**	0.013*
**V/Cre**	**2.9 (1.7–8.2)**	**6.7 (2.8–12.8)**	0.022*
**Fe/Cre**	**15 (11–25)**	**22 (13–55)**	0.030*
**Rb/Cre**	2035 (1453–2577)	1702 (1199–2378)	0.099
**Ni/Cre**	9.5 (5.2–13.5)	8.2 (4.3–10.6)	0.13
**Sr/Cre**	100 (63–134)	113 (74–185)	0.21
**Mn/Cre**	0.19 (0.09–0.43)	0.31 (0.16–0.52)	0.24
**Cd/Cre**	0.099 (0.059–0.16)	0.090 (0.042–0.14)	0.36
**Cr/Cre**	0.75 (0.37–1.00)	0.70 (0.41–1.74)	0.36
**Zn/Cre**	253 (145–182)	204 (124–332)	0.40
**Ba/Cre**	8.6 (5.9–12.6)	8.4 (4.5–12.6)	0.43

*Denotes significance using the Benjamini-Hochberg procedure with a 0.1 false discovery rate.

IQR = Interquartile range

**Table 3:** Reduced multivariable regression models for the effects of CaOx stone status and environmental factors on log-transformed urinary element-to-creatinine ratios (Ca/Cre, Co/Cre, Cu/Cre, Fe/Cre and V/Cre). For variables with 1 degree of freedom, the status corresponding to the estimate is in parentheses.

	Estimate	Standard Error	T value	Degrees of Freedom	P-value
**Ca/Cre**					
				2	**8.7E-07**
Stone status (case)	0.82	0.15	5.58	1	**1.5E-07**
Sex (male)	-0.33	0.16	-2.12	1	**0.036**
**Co/Cre**					
Stone status (case)	-0.39	0.18	-2.19	1	**0.030**
**Cu/Cre**					
				5	**0.0060**
Stone status (case)	0.44	0.13	3.26	1	**0.0015**
Age	0.06	0.03	2.21	1	**0.029**
Breed				3	0.081
Miniature Schnauzer	0.32	0.16	2.09		**0.039**
Bichon Frise	0.18	0.19	0.96		0.34
Shih Tzu	-0.12	0.22	-0.54		0.59
**Fe/Cre**					
Stone status (case)	0.44	0.18	2.4	1	**0.016**
**V/Cre**					
				2	**0.038**
Stone status (case)	0.50	0.22	2.23	1	**0.027**
Log(weight)	-0.44	0.31	-1.42	1	**0.16**

The medical history for the dogs with the 10 highest values for urinary Ca/Cre, Cu/Cre, Fe/Cre, and V/Cre were examined to determine if there was any overt shared characteristic or exposure history (diet or medication). The 10 highest urinary Ca/Cre values were all from male CaOx cases. Breeds included 5 Miniature Schnauzers, 3 Bichons Frise, 2 Lhasa Apsos, and a mixed breed dog. Three dogs were fed Hill’s u/d, two were fed Royal Canin SO, and the others were consuming five diverse diets. Medications included doxycycline (1), tramadol (1), and carprofen (1). The top 10 urinary Cu/Cre values were from 7 male and 3 female dogs with 5 CaOx cases and 5 controls. Breeds included 7 Miniature Schnauzers, 1 Bichon Frise, 1 Yorkshire Terrier, and a mixed breed dog. Two dogs were fed Royal Canin SO, and the others were consuming seven diverse diets. One dog was receiving daily supplements (GNC Pets Ultra Mega Hip & Joint Health for Senior Dogs and Ultra Mega Multivitamin Plus for Adult Dogs). The 10 highest urinary Fe/Cre values came from 9 male and 1 female CaOx cases. Breeds included 4 Miniature Schnauzers, 2 Shih Tzus, 1 Bichon Frise, 1 Fox Terrier, 1 Rat Terrier, and a mixed breed dog. One dog was consuming Royal Canin SO, and the others were consuming nine diverse diets. Medications included doxycycline (1, the dog mentioned above) and amoxicillin/clavulanic acid (1). The 10 highest urinary V/Cre values came from 6 male and 4 female dogs with 6 CaOx cases and 4 controls. Breeds included 5 Miniature Schnauzers, and 1 each of Bichon Frise, Chihuahua, Lhasa Apso, Pomeranian, and Standard Schnauzer. Two dogs were consuming Hill’s u/d, one was consuming Royal Canin SO, and the others were consuming seven diverse diets. One dog was being administered cyclosporine ophthalmic drops.

### Correlations between urinary metals

Correlation coefficients were calculated for the urinary metals and are presented in Table 4. Urinary Ca/Cre has a strong positive correlation (r = 0.7) with Sr/Cre, moderate positive correlations (r = 0.3) with urinary Cr/Cre, Ni/Cre, and Zn/Cre, and weak positive correlations with other metals.

**Table 4:** Heat map of correlation coefficients between urinary metals in dogs with and without CaOx urolithiasis. Correlation coefficients in bold font had p-values < 0.05.

	Ca/Cre	Sr/Cre	Cr/Cre	Ni/Cre	Zn/Cre	Ba/Cre	Cu/Cre	Mn/Cre	Rb/Cre	Cd/Cre	V/Cre	Fe/Cre	Co/Cre
Ca/Cre													
Sr/Cre	**0.7**												
Cr/Cre	**0.3**	**0.3**											
Ni/Cre	**0.3**	**0.3**	**0.4**										
Zn/Cre	**0.3**	**0.3**	**0.4**	**0.6**									
Ba/Cre	**0.2**	**0.5**	**0.2**	**0.2**	**0.4**								
Cu/Cre	**0.2**	0.1	**0.2**	0.1	**0.3**	0.2							
Mn/Cre	**0.2**	**0.2**	**0.3**	0.2	**0.5**	**0.5**	**0.3**						
Rb/Cre	**0.2**	**0.2**	0.2	**0.3**	**0.3**	0.2	**0.4**	0.2					
Cd/Cre	0.1	**0.2**	**0.5**	**0.4**	**0.5**	**0.3**	**0.2**	**0.4**	**0.3**				
V/Cre	0.1	0	**0.4**	**0.2**	**0.2**	0	0.1	0.1	0	0.1			
Fe/Cre	0.1	0	**0.3**	0.1	**0.4**	0.1	**0.3**	**0.4**	**0.2**	**0.2**	0		
Co/Cre	0.1	**0.3**	**0.2**	**0.4**	**0.2**	0.2	**0.2**	0.1	**0.2**	**0.3**	0	0.1	

## Discussion

The present study describes relationships between urinary metal concentrations and CaOx urolithiasis in a spontaneous canine model. While the design of this study does not permit causality to be evaluated, it provides data to stimulate further research into the role of metals in stone disease. Many of the relationships identified in this study parallel findings in human CaOx stone formers and thus highlight the suitability of the canine model.

Hypercalciuria is the most common metabolic abnormality underlying urolithiasis in people [7] and occurs in canine CaOx stone formers as well [10, 22, 23]. Approximately two thirds of the study population was comprised of three breeds, the Bichon Frise, Miniature Schnauzer, and Shih Tzu, included in a previous study demonstrating idiopathic hypercalciuria in each of these breed [10]. In line with this, urinary Ca/Cre was significantly higher in CaOx cases in this study. Unexpectedly, male sex had a negative association with Ca/Cre in a multivariable regression analysis. Men have higher daily urinary calcium excretion than women, but this difference has been attributed to body weight [24]; weight was included in the multivariate regression analysis for urinary calcium in the present study and did not have a significant effect. The previous canine study that included the aforementioned breed-matched subset of the current study population found no relationship between sex and urinary Ca/Cre [10]. The finding of lower urinary Ca/Cre in male dogs in this study warrants further investigation.

In this study, urinary Fe/Cre was significantly higher in dogs with CaOx urolithiasis. This finding was not explained by age, sex, or the diet categories evaluated. Hematuria can occur with urolithiasis and would presumably increase urinary iron. Urine sediment was not examined in this study, but median urinary Fe/Cre did not differ between dogs that had stones at the time of urine collection compared to those with historic urolithiasis. Iron is the third most abundant metal in human kidney stones, after zinc and strontium, and is more abundant in calcium-containing stones than other stone types [14]. Iron has also been detected in canine CaOx stones [15]. Iron overload is linked to hypercalciuria in animal models and people and is believed to be due to increased bone turnover [25, 26]. However, urinary Fe/Cre was not correlated with urinary Ca/Cre in this study. An alternative explanation is that Fe has an effect on CaOx urolithiasis via other mechanisms. A study of human idiopathic CaOx kidney stone formers found that the mean urinary Fe was more than 30 times greater in these patients compared with controls, despite lower serum iron concentrations [27]. Ferric ions and citrate are inhibitors of CaOx crystal development *in vitro*, but when present together, their inhibitory effects diminish due to formation of iron-citrate complexes that lack inhibitory activity [28]. Thus, high urinary iron could theoretically increase risk for stone formation via limiting the free citrate available to bind calcium and prevent crystallization.

It is noteworthy that one of the common therapeutic stone-prevention diets (Hill’s u/d) had a negative correlation with urinary Fe/Cre. This diet was only prescribed after stone diagnosis, and the cases on the diet may have previously had a higher urinary Fe/Cre. Thus, the association between urinary iron and stone risk could be greater than estimated by this data. A more in-depth discussion of this limitation is included later.

The CaOx cases also had higher urinary Cu/Cre. Trace concentrations of copper have been reported in human and canine CaOx stones [14, 15, 29], but the relationship between urinary copper and CaOx urolithiasis is unclear with conflicting results across human studies [27, 30, 31]. In this study, though the median Cu/Cre was higher in CaOx cases, the 10 highest measurements were evenly distributed among cases and controls. Urinary Cu/Cre had a weak correlation with urinary Ca/Cre (r = 0.2). Copper may inhibit calcium phosphate crystallization but is not believed to have a direct effect on CaOx crystallization [13].

Urinary V/Cre was higher in CaOx cases compared to controls; however, further analysis revealed a strong positive relationship with the therapeutic stone-prevention diet Hill’s u/d and a trend with Royal Canin SO. Neither of these diets report vanadium content, but brewers rice is the top ingredient in both. Rice has a moderate to high vanadium content [32, 33] and may be the explanation for the association of higher urinary V/Cre in dogs eating these diets.

In contrast to the other metals that differed between cases and controls, urinary Co/Cre was lower in CaOx cases than controls. Cobalt has been detected in human CaOx stones, but it is also present in other stone types and had no correlation with stone composition [34]. Cobalt may inhibit CaOx crystallization *in vitro* [13], but limited data is available on the role of this metal in lithogenesis. Urinary Sr/Cre was found to have a strong correlation with Ca/Cre (r = 0.7), though it was significantly different between CaOx cases and controls. This finding matches data from previous literature. Strontium is the second most abundant metal detected in human kidney stones and Randall’s plaques [14, 35, 36]. Strontium has a similar size and charge to calcium, and its absorption and excretion follow that of calcium [37]. In fact, oral strontium loading is used to study calcium metabolism in patients with idiopathic hypercalciuria [38, 39]. To the authors’ knowledge, a direct role of strontium in lithogenesis has not been reported.

Unlike the other metals analyzed, cadmium is an environmental toxin with a well-documented effect on urolithiasis risk. It accumulates in renal tubular cells and causes hypercalciuria through inhibition of calcium resorption, effects on calcium uptake by bone, and alterations in vitamin D metabolism [16, 40]. Low level exposure is associated with increased stone risk without other classic signs of toxicity [17]. Renal tissue accumulates cadmium, and it is very slowly eliminated through the urine. Urinary cadmium is therefore considered a biomarker of lifetime cadmium exposure [41]. Toxicokinetic studies of chronic oral cadmium dosing in dogs have estimated the biological half-life at 1–2 years for a two compartment model, and human data suggests that it may take 10–30 years for elimination from the renal cortex and other tissues [42, 43]. Evidence of cadmium toxicity was not present in the urine of the dogs in this study. Future studies involving greater numbers of dogs from diverse regions of the country are needed to determine whether cadmium is a substantial environmental risk factor for canine CaOx urolithiasis.

As stated above, one important limitation of this study is that the cross-sectional design does not permit determination of the temporal relationship between metals and CaOx urolithiasis or whether they are direct relationships or the result of a mediator variable. Urine samples were obtained from the majority of the cases at the time that a stone was present, but some dogs had been diagnosed with stones more than a year prior to study enrollment. The time gap between stone formation and the urine sample complicates the ability to determine the source of the metal exposure and when the exposure occurred relative to stone formation. A metal exposure may have been missed if the metal was eliminated from the body before the urine sample was obtained. This is most concerning for elements that are tightly regulated by the body and have short elimination half-lives, such as calcium, zinc, and iron. Over a third of the cases were on therapeutic stone-prevention diets formulated to reduce the urinary CaOx relative supersaturation that were not prescribed until after one or more stone episodes. As such, diet is an example of an exposure that changed for some dogs between stone formation and study enrollment and could confound the analyses. Diet was included in the regression models for the cases and, as described above, one of the therapeutic stone-prevention diets was associated with urinary iron and vanadium. Detailed diet histories and analyses were not performed beyond the classification described, and we may have missed other relationships between nutrients and urinary metals.

Other limitations pertain to the population of dogs studied. Most of the dogs in this study were of three breeds. While breed was considered as a covariate in the regression analyses, it was classified into only four categories, the three top breeds and “other.” The “other” category comprised 25 dogs of 16 breeds plus 12 mixed breed dogs. There could be breed differences in the pathophysiology of stone formation, and the study was not designed to enable detection of breed-specific associations between urinary metals and CaOx stone disease outside of the three top breeds included. The cases and controls were also not sex- and age-matched, but these variables were included as predictors in the regression models. The study took place at the University of Minnesota, and all dogs were local to Minnesota and its surrounding states. There may be geographical differences in risk factors for CaOx stone disease, and the findings of this study may not be applicable to the general canine population.

There are also some limitations to the methodology. Twenty-four hour urine collection is a considered superior method of screening for hypercalciuria than a fasting spot UCa/Cr in people [44–46], and 24-hour samples are commonly used to quantify other urinary metals as well [27, 30–31, 34]. The authors are not aware of any direct comparisons of spot versus 24-hour urinary element measurements in dogs, and both methods have been used to demonstrate the presence of hypercalciuria in dogs with CaOx urolithiasis [10, 47–49]. Spot samples were used in this study due to the convenience, technical ease, and lower expense compared with complete urine collection in dogs. Finally, the study used a preset panel for metal analysis. Other metals not included on the panel could have an important relationship to CaOx stones.

### Conclusions

This study used a spontaneous canine model to identify associations between urinary metals and CaOx urolithiasis. The CaOx cases had hypercalciuria, the most common metabolic abnormality in people with CaOx stones. The cases also had higher urinary iron, which again parallels human data. *In vitro* studies suggest that iron can bind citrate and create complexes that diminish the inhibitory activity of citrate on crystallization. However, a direct role of iron in lithogenesis has not been established. Higher copper and vanadium and lower urinary cobalt were also present in the CaOx cases. The higher urinary vanadium was linked to a therapeutic stone-prevention diet. The significance of the other metals in relation to stone formation is unknown. Further investigation into the role of metals in stone formation could shed light on the pathogenesis of CaOx urolithiasis and have implications for nutritional recommendations.

## Supporting information

S1 File Detailed clinical and urinary data for the 122 study dogs. (XLSX)

S1 Table Multivariable regression models for the effects of CaOx stone status and environmental factors on urinary element-to-creatinine ratios. (DOCX)

S2 Table Multivariable regression models for the effects of diet and active stone disease on urinary element-to-creatinine ratios in dogs with CaOx urolithiasis. (DOCX)
