# Supplementary material for: Urinary metals in a spontaneous canine model of calcium oxalate urolithiasis
Source: PLoS One. 2017 May 3;12(5):e0176595. doi: 10.1371/journal.pone.0176595 (PMC5415176; doi:10.1371/journal.pone.0176595)
Supplement: S1 Table — (DOCX) [file pone.0176595.s002.docx]

**S1 Table.** Multivariable regression and analysis of covariance (ANCOVA) results for the effects of CaOx stone status and environmental factors on log-transformed urinary element-to-creatinine ratios (Ca/Cre, Co/Cre, Cu/Cre, Fe/Cre and V/Cre). For variables with 1 degree of freedom, the status corresponding to the estimate is in parentheses.

|  | **Estimate** | **Standard Error** | **T value** | **Degrees of Freedom** | **P-value** |
| --- | --- | --- | --- | --- | --- |
| **Ca/Cre** |  |  |  |  |  |
|  | |  |  | 7 | **6.4E-05** |
| Stone status (case) | 0.79 | 0.16 | 4.85 | 1 | **4.0E-06** |
| Sex (male) | -0.25 | 0.18 | -1.43 | 1 | 0.15 |
| Age | 0.01 | 0.03 | 0.28 | 1 | 0.78 |
| Breed |  |  |  | 3 | 0.53 |
| Miniature Schnauzer | -0.16 | 0.18 | -0.900 |  | 0.37 |
| Bichon Frise | 0.03 | 0.22 | 0.11 |  | 0.91 |
| Shih Tzu | 0.12 | 0.27 | 0.44 |  | 0.66 |
| Log(weight) | -0.23 | 0.22 | -1.01 | 1 | 0.31 |
| **Co/Cre** |  |  |  |  |  |
|  | |  |  | 7 | 0.28 |
| Stone status (case) | -0.28 | 0.21 | -1.38 | 1 | 0.17 |
| Sex (male) | -0.09 | 0.22 | -0.42 | 1 | 0.67 |
| Age | 0.04 | 0.04 | 0.94 | 1 | 0.35 |
| Breed |  |  |  | 3 | 0.67 |
| Miniature Schnauzer | 0.17 | 0.23 | 0.72 |  | 0.47 |
| Bichon Frise | -0.10 | 0.28 | -0.35 |  | 0.73 |
| Shih Tzu | -0.11 | 0.34 | -0.34 |  | 0.74 |
| Log(weight) | -0.31 | 0.28 | -1.11 | 1 | 0.27 |
| **Cu/Cre** |  |  |  |  |  |
|  | |  |  | 7 | **0.018** |
| Stone status (case) | 0.45 | 0.14 | 3.19 | 1 | **0.0018** |
| Sex (male) | -0.05 | 0.15 | -0.35 | 1 | 0.72 |
| Age | 0.06 | 0.03 | 2.19 | 1 | **0.031** |
| Breed |  |  |  | 3 | 0.10 |
| Miniature Schnauzer | 0.33 | 0.16 | 2.07 |  | **0.041** |
| Bichon Frise | 0.19 | 0.19 | 1.00 |  | 0.32 |
| Shih Tzu | -0.17 | 0.19 | -0.37 |  | 0.71 |
| Log(weight) |  |  |  | 1 | 0.38 |
|  |  |  |  |  |  |
| **S1 Table (continued)** | | | | | |
|  | **Estimate** | **Standard Error** | **T value** | **Degrees of Freedom** | **P-value** |
| **Fe/Cre** |  |  |  |  |  |
|  | |  |  | 7 | 0.10 |
| Stone status (case) | 0.59 | 0.20 | 2.86 | 1 | **0.0050** |
| Sex (male) | -0.23 | 0.23 | -1.01 | 1 | 0.32 |
| Age | 0.06 | 0.04 | 1.56 | 1 | 0.12 |
| Breed |  |  |  | 3 | 0.32 |
| Miniature Schnauzer | 0.20 | 0.23 | 0.87 |  | 0.38 |
| Bichon Frise | -0.23 | 0.28 | -0.82 |  | 0.42 |
| Shih Tzu | -0.19 | 0.34 | -0.56 |  | 0.57 |
| Log(weight) | 0.16 | 0.28 | 0.57 | 1 | 0.57 |
| **V/Cre** | |  |  |  |  |
|  | |  |  | 7 | 0.21 |
| Stone status (case) | 0.39 | 0.26 | 1.54 | 1 | 0.13 |
| Sex (male) | 0.34 | 0.28 | 1.23 | 1 | 0.22 |
| Age | -0.01 | 0.05 | -0.16 | 1 | 0.87 |
| Breed |  |  |  | 3 | 0.67 |
| Miniature Schnauzer | -0.14 | 0.29 | -0.48 |  | 0.63 |
| Bichon Frise | 0.26 | 0.35 | 0.73 |  | 0.47 |
| Shih Tzu | -0.03 | 0.42 | -0.07 |  | 0.95 |
| Log(weight) | -0.61 | 0.35 | -1.73 | 1 | 0.086 |
