# Supplementary material for: Urinary metals in a spontaneous canine model of calcium oxalate urolithiasis
Source: PLoS One. 2017 May 3;12(5):e0176595. doi: 10.1371/journal.pone.0176595 (PMC5415176; doi:10.1371/journal.pone.0176595)
Supplement: S2 Table — (DOCX) [file pone.0176595.s003.docx]

**S2 Table.** Multivariable regression models for the effects of diet and active stone disease on log-transformed urinary element-to-creatinine ratios (Ca/Cre, Co/Cre, Cu/Cre, Fe/Cre and V/Cre) in dogs with CaOx urolithiasis. For variables with 1 degree of freedom, the status corresponding to the estimate is in parentheses.

|  | | | **Estimate** | | **Standard Error** | | **T value** | **Degrees of Freedom** | **P-value** |
| --- | --- | --- | --- | --- | --- | --- | --- | --- | --- |
| **Ca/Cre** | | |  | |  | |  |  |  |
|  |  | | |  | | | | 3 | 0.36 |
| Active disease (yes) | | | -0.07 | | 0.25 | | -0.27 | 1 | 0.79 |
| Diet | | |  | |  | |  | 2 | 0.21 |
| Hill’s u/d | | | 0.38 | | 0.24 | | 1.62 |  | 0.11 |
| Royal Canin SO | | | 0.33 | | 0.29 | | 1.12 |  | 0.27 |
| **Co/Cre** | | |  | |  | |  |  |  |
|  |  | | |  | | | | 3 | 0.71 |
| Active disease (yes) | | -0.11 | | 0.26 | | -0.39 | | 1 | 0.70 |
| Diet | |  | |  | |  | | 2 | 0.53 |
| Hill’s u/d | | 0.00 | | 0.26 | | 0.01 | |  | 0.99 |
| Royal Canin SO | | 0.35 | | 0.32 | | 1.11 | |  | 0.27 |
| **Cu/Cre** | | |  | |  | |  |  |  |
|  |  | | |  | | | | 3 | 0.22 |
| Active disease (yes) | | -0.33 | | 0.18 | | -1.87 | | 1 | 0.065 |
| Diet | |  | |  | |  | | 2 | 0.49 |
| Hill’s u/d | | 0.08 | | 0.17 | | 0.46 | |  | 0.65 |
| Royal Canin SO | | 0.25 | | 0.21 | | 1.18 | |  | 0.24 |
| **Fe/Cre** | | |  | |  | |  |  |  |
|  |  | | |  | | | | 3 | 0.033 |
| Active disease (yes) | | -0.11 | | 0.32 | | -0.34 | | 1 | 0.73 |
| Diet | |  | |  | |  | | 2 | 0.015 |
| Hill’s u/d | | -0.90 | | 0.31 | | -2.96 | |  | **0.0042** |
| Royal Canin SO | | -0.41 | | 0.38 | | -1.10 | |  | 0.27 |
| **V/Cre** |  | | |  | | | |  |  |
|  |  | | |  | | | | 3 | 0.12 |
| Active disease (yes) | | -0.05 | | 0.35 | | -0.16 | | 1 | 0.88 |
| Diet | |  | |  | |  | | 2 | 0.055 |
| Hill’s u/d | | 0.74 | | 0.34 | | 2.19 | |  | **0.032** |
| Royal Canin SO | | 0.66 | | 0.42 | | 1.59 | |  | 0.12 |
